# Global efforts to identify and support people with tuberculosis

**DOI:** 10.1186/s44263-024-00067-0

**Published:** 2024-05-29

**Authors:** Gerrit John-Schuster

**Affiliations:** Springer Nature, BioMed Central, New York, NY USA

**Keywords:** Tuberculosis, Epidemiology, Global health

*We are pleased to present the content in one of our first collections, focused on identifying people with tuberculosis (TB) and linking to care. Guided by our guest editors, the collection has attracted studies covering timely and diverse topics that are of relevance even beyond TB.*

Tuberculosis (TB) remains a leading cause of death from infectious diseases, where global and public health efforts are needed to find the millions missed each year and achieve the three pillars of the End TB Strategy (i.e. reducing TB deaths, TB incidence, and catastrophic costs faced by TB-affected families due to TB) [1]. Major research activities in the areas of TB diagnosis, incidence and prevalence, and implementation of early detection, prevention, treatment, and care contribute to advancing these goals.

As timely diagnosis is key to eliminating any disease, the development of novel or improved approaches as well as their application in remote areas might help in reaching all populations at risk and not just those with full and immediate access to healthcare and testing. Globally delivered community-based active case finding (ACF) for TB, a rather holistic approach that entails a range of activities including screening of at-risk populations, health promotion, community engagement, and health service strengthening, might significantly alter TB epidemiology. However, for these to be effective and show a population-level impact, ACF programs must be carefully planned and delivered. This should include giving attention to the selection of populations, screening algorithms, selecting outcomes, and monitoring and evaluation, as summarized by MacPherson and colleagues in a Review published in this collection [2]. Cost-effectiveness of ACF is another important point to consider. Dowdy & Sohn advocate for a consensus framework for estimating the long-term cost and impact of ACF [3], including estimates of which costs to measure; prevented morbidity, mortality, and transmission; measurable short-term outcomes; and meaningful cost-effectiveness thresholds.

Technological advances will be similarly important for timely diagnosis of TB, including the use of artificial intelligence (AI) for example for chest X-ray (CXR) interpretation. Creswell and colleagues highlight early user experiences from nine high TB-burden countries. The authors provide practical considerations and best practices related to deployment, threshold and use case selection, and scale-up, with the ultimate goal of enhancing access to this innovative technology [4]. An application of AI-supported CXR interpretation was presented by John et al. [5] who compared the use of ultra-portable devices to symptom screening in an ACF campaign in Nigeria. The authors found that ultra-portable CXR provided more efficient TB screening in hard-to-reach areas, as symptom screening missed large proportions of people with bacteriologically confirmed TB in their study.

Another pressing topic is how to identify people with subclinical TB, i.e., those with microbiological evidence of disease caused by *Mycobacterium tuberculosis*, but who either do not have or do not report TB symptoms. This can significantly impact TB transmission and is especially relevant for people sharing living quarters, such as people who are incarcerated or who are sharing a household [6]. However, data on this are still scarce. In this collection, Carter et al. estimated the prevalence of subclinical pulmonary TB in household contacts of index TB patients in two South African provinces, and how this differed by HIV status [7]. The authors found that almost 71% of pulmonary TB diagnosed in household contacts in this setting was subclinical. Additionally, subclinical TB prevalence was higher in people living with HIV compared to those who were HIV-negative.

Children are another group of people who are affected by TB but often not diagnosed. As microbial diagnosis of pulmonary TB requires a sputum sample, this is especially challenging in young children who cannot spontaneously expectorate. A more easily collected sample in children could be nasopharyngeal aspirates (NPA). Their diagnostic yield for testing by either culture or nucleic acid amplification testing was systematically reviewed and meta-analyzed by Khambati et al. [8], which confirmed NPA as a suitable and feasible approach for diagnosing pediatric TB. Nevertheless, more research is needed to optimize and standardize this method for improved diagnostic yield.

Dauphinais and colleagues raise awareness of an often neglected key TB determinant and comorbidity: malnutrition, which is a cause of secondary immunodeficiency and has been termed nutritionally acquired immunodeficiency syndrome (N-AIDS) [9]. The authors call for special consideration of malnutrition in TB elimination efforts. They envision that addressing this modifiable risk factor will help in TB detection, treatment, and prevention. Howell et al. specifically address this topic by presenting an evaluation of a results-based financing nutrition intervention for TB patients in India, which was implemented during the challenging COVID-19 pandemic [10].

Finally, more research into the preferences of people and communities most affected by TB are needed in order to inform and improve screening and testing services. This will be paramount for understanding the acceptability, accessibility, and appropriateness of TB detection strategies among hard-to-reach and yet-to-be-diagnosed populations. Kerkhoff and colleagues provide an overview of qualitative preference exploration and quantitative preference elicitation research methods as well as key opportunities (i.e., most preferred features of novel tools; resonant communication strategies; setting-specific barriers and facilitators to screening and testing, and most valued features of detection and treatment strategies) that will assist in moving this area forward [11].

As the breadth of the topics covered in this collection indicates, there is still a lot to be learned, studied, improved, and implemented to ensure that everyone with or exposed to TB is found, diagnosed, and linked to care.

## Data Availability

Not applicable.
